# Journaling with large language models: a novel UX paradigm for AI-driven personal health management

**DOI:** 10.3389/frai.2025.1567580

**Published:** 2025-06-24

**Authors:** Birger Moëll, Fredrik Sand Aronsson

**Affiliations:** ^1^Division of Speech, Music and Hearing, School of Electrical Engineering and Computer Science, KTH Royal Institute of Technology, Stockholm, Sweden; ^2^Division of Speech and Language Pathology, Department of Clinical Science, Intervention and Technology, Karolinska Institutet, Stockholm, Sweden; ^3^Theme Womens Health and Allied Health Professionals, Section of Speech and Language Pathology, Karolinska University Hospital, Stockholm, Sweden

**Keywords:** large language models (LLMs), AI-driven journaling, patient engagement, health literacy, explainable AI, data privacy, natural language processing (NLP), medical AI

## Abstract

**Introduction:**

The integration of large language models (LLMs) into personal health management presents transformative potential, but faces critical challenges in user experience (UX) design, ethical implementation, and clinical integration.

**Method:**

This paper introduces a novel AI-driven journaling application, a functional prototype available open source, designed to encourage patient engagement through a natural language interface. This approach, termed “AI-assisted health journaling,” enables users to document health experiences in their own words while receiving real-time, context-aware feedback from an LLM. The prototype combines a personal health record with an LLM assistant, allowing for reflective self-monitoring and aiming to combine patient-generated data with clinical insights. Key innovations include a three-panel interface for seamless journaling, AI dialogue, and longitudinal tracking, alongside specialized modes for interacting with simulated healthcare expert personas.

**Result:**

Preliminary insights from persona-based evaluations highlight the system's capacity to enhance health literacy through explainable AI responses while maintaining strict data localization and privacy controls. We propose five design principles for patient-centric AI health tools: (1) decoupling core functionality from LLM dependencies, (2) layered transparency in AI outputs, (3) adaptive consent for data sharing, (4) clinician-facing data summarization, and (5) compliance-first architecture.

**Discussion:**

By transforming unstructured patient narratives into structured insights through natural language processing, this approach demonstrates how journaling interfaces could serve as a critical middleware layer in healthcare ecosystems-empowering patients as active partners in care while preserving clinical oversight. Future research directions emphasize the need for rigorous trials evaluating impacts on care continuity, patient-provider communication, and long-term health outcomes across diverse populations.

## 1 Introduction

The integration of Large Language Models (LLMs) into healthcare systems represents a paradigm shift in how patients interact with medical information and manage their health. While LLMs like GPT-4 have demonstrated remarkable capabilities in clinical reasoning and patient education (Singhal et al., 2023), their deployment in real-world health contexts remains constrained by critical challenges: ensuring reliable outputs, maintaining patient trust, and aligning with clinical workflows (Abd-alrazaq et al., 2023; Nassiri and Akhloufi, 2024).

For the purposes of this paper, we define AI-assisted health journaling as the process of an individual regularly recording their health status, symptoms, experiences, and reflections in a digital format, where an LLM provides interactive feedback, insights, and summaries based on these entries. This differs from simple digital note-taking by incorporating an AI layer that actively processes and responds to the user's narrative content to support self-management and understanding.

Recent advances in local LLMs that operate entirely on-device present new opportunities to address these challenges while enhancing privacy protections. Models like DeepSeek R1 (DeepSeek-AI et al., 2025) demonstrate that performant LLMs can run locally on consumer hardware without cloud dependencies, enabling real-time processing of sensitive health data while maintaining complete data sovereignty. This on-device processing aligns with emerging regulatory frameworks like the EU AI Act that emphasize data minimization and local processing for health applications (European Parliament and Council of the European Union, 2024). If model capabilities keep improving at a similar rate, on-device medical LLMs will become a reality, and with functional local LLMs, many hurdles for the integration of AI in healthcare will dissolve.

Traditional electronic health records (EHRs), while transformative for clinical documentation, frequently fail to capture the richness of patients' lived experiences (Varadarajan and Dutta, 2024). This “narrative gap” in healthcare data–the absence of contextual details about symptom evolution, lifestyle factors, and treatment adherence–limits clinicians' ability to deliver personalized care (Varadarajan and Dutta, 2024). Patient-generated health data (PGHD) through journaling has shown promise in bridging this gap, particularly in chronic disease management (Treadwell et al., 2021), but existing tools often suffer from poor usability (Treadwell et al., 2021) or lack sophisticated analytical capabilities.

We propose that LLM-augmented journaling systems can address these challenges by creating a dynamic interface for continuous patient engagement. Unlike conventional symptom trackers, our systems leverage three key LLM capabilities:

1. Natural language understanding of unstructured patient narratives.
2. Contextual memory for longitudinal health patterns.
3. Adaptive communication styles that build health literacy.

When grounded in personal health records (PHRs), this approach enables what we term “reflective self-monitoring”—a process where patients not only record health data but actively interpret it through AI-mediated dialogue. For any health issue, there is often both a somatic and a psychological component. We believe that AI health journaling can be helpful for both improving autonomy and understanding of health while potentially mitigating health anxiety.

Our case study presents a novel PHR architecture that embeds LLM capabilities within a journaling interface while addressing core ethical and regulatory concerns. This paper describes a functional prototype of such a system available fully open source. The following sections detail our system architecture, present use cases derived from a preliminary qualitative evaluation using patient personas, and discuss implications for healthcare ecosystems. We conclude with a roadmap for clinical validation and regulatory compliance, emphasizing the need for continuous alignment between AI capabilities and patient-centered care paradigms.

## 2 Materials and methods

### 2.1 AI health journaling as a new user interface paradigm in healthcare

Health journaling, enhanced with AI, emerges as an especially promising interface for LLMs due to its inherent characteristics and potential benefits within the healthcare landscape. This paradigm offers a unique blend of traditional self-reflection with modern AI capabilities:

- **Coexistence with traditional healthcare channels:** AI-assisted journaling can supplement existing communication methods such as chat, phone, and in-person visits without disrupting established clinical workflows. It acts as an additional data source and engagement tool.
- **Encouragement of reflection:** The act of journaling encourages thoughtful, longitudinal input, making it less likely that users will solely rely on it for immediate medical advice for acute, urgent conditions, distinguishing it from on-demand diagnostic AI tools.
- **Functional independence from LLMs:** A well-designed journaling application remains useful for record-keeping even if the LLM component is disabled or limited. This modularity simplifies compliance with regulatory requirements and allows for revoking or restricting AI access if necessary.
- **Support for privacy and local data storage:** User data can be stored on their personal devices. The advent of capable on-device LLMs further enhances this, allowing sensitive information to be processed locally, significantly improving privacy and data sovereignty.
- **Enhancement of clinical utility:** Health journaling is already a common practice in managing chronic diseases and in mental health care (Sohal et al., 2022; Boulter, 2024). Integrating LLMs can structure these narratives, extract key information, and potentially highlight trends, thus creating a richer data source for healthcare providers.
- **Enablement of seamless data sharing:** While maintaining user control, journaling data can be formatted and summarized for integration into EHR systems, potentially improving clinical decision-making and patient outcomes through a more comprehensive patient history.
- **Adaptability to strict regulations:** By decoupling the core journaling functionality from AI components, developers can more easily navigate and align with the evolving landscape of healthcare and AI regulations. Prompt engineering can be employed to ensure AI responses are supportive and informational, rather than prescriptive medical advice.
- **Provision of evolving AI benefits:** As LLM technology advances, new features (e.g., improved summarization, pattern recognition, educational content delivery) can be integrated into the interface, provided they are deemed safe, effective, and ethically sound.

Building on these advantages, a journaling-based approach to LLM integration offers a flexible, user-centric, and ethically considerate model for future healthcare applications. Based on these reasons, we chose to build a standalone journaling application for healthcare that incorporates the use of LLMs. We believe that this choice of interface can be paramount in the future of AI in healthcare, as it offers an easy-to-use, patient-centric way to access LLMs in a relatively low-risk, supportive scenario.

**Figure 1:**
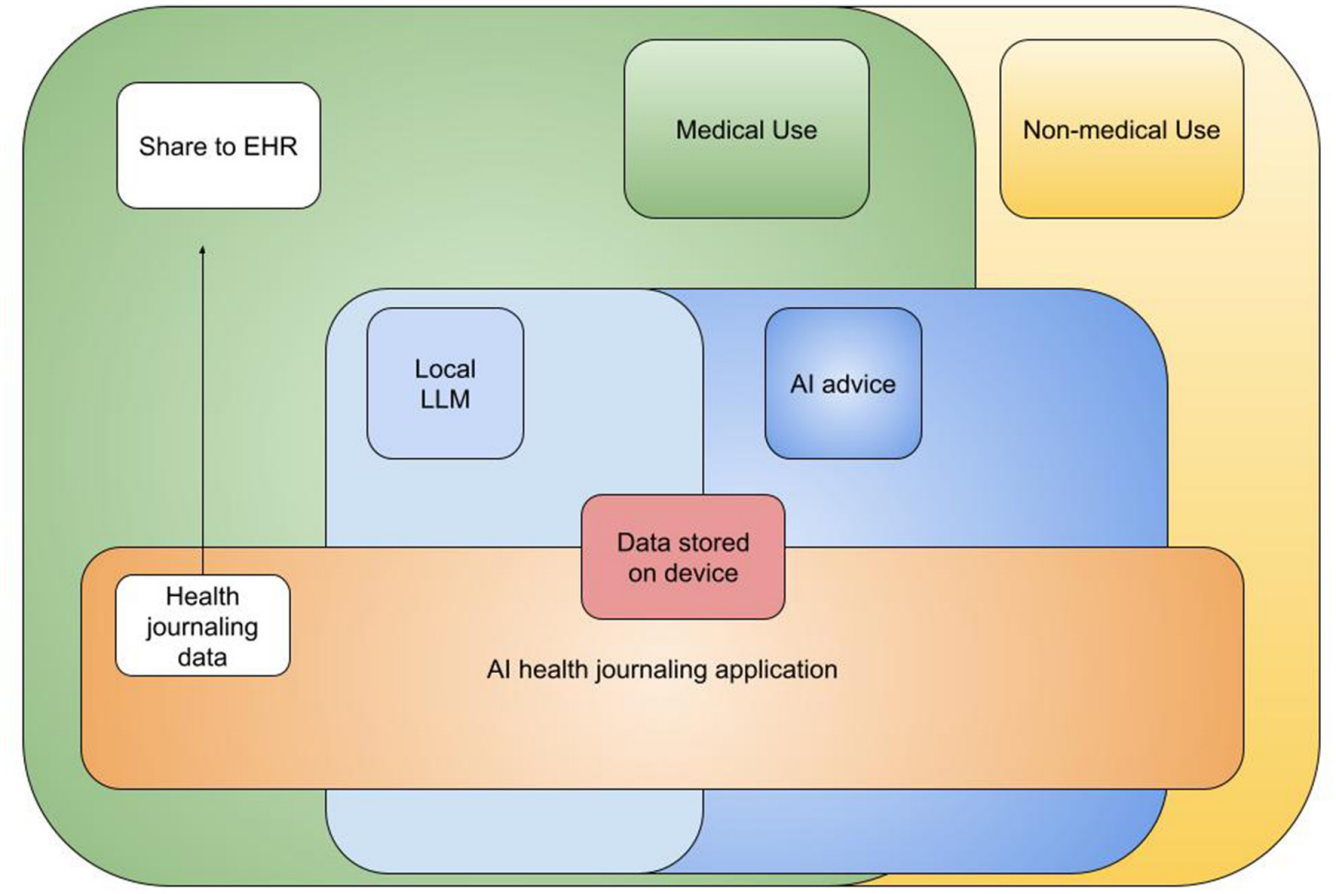
Overview of the AI journaling app's conceptual role and integration within the broader healthcare system, illustrating its function as a bridge between patient self-management and formal healthcare services.

Figure 1 outlines how the app is conceptualized to integrate into the current healthcare system, where the journaling application interfaces with existing systems like EHRs while also functioning as a standalone system for both medical and non-medical wellness use cases. This paper outlines our work in building the functional prototype of this interface and our thoughts on how it can contribute to the future of healthcare infrastructure.

### 2.2 Overview of the interface

**Figure 2:**
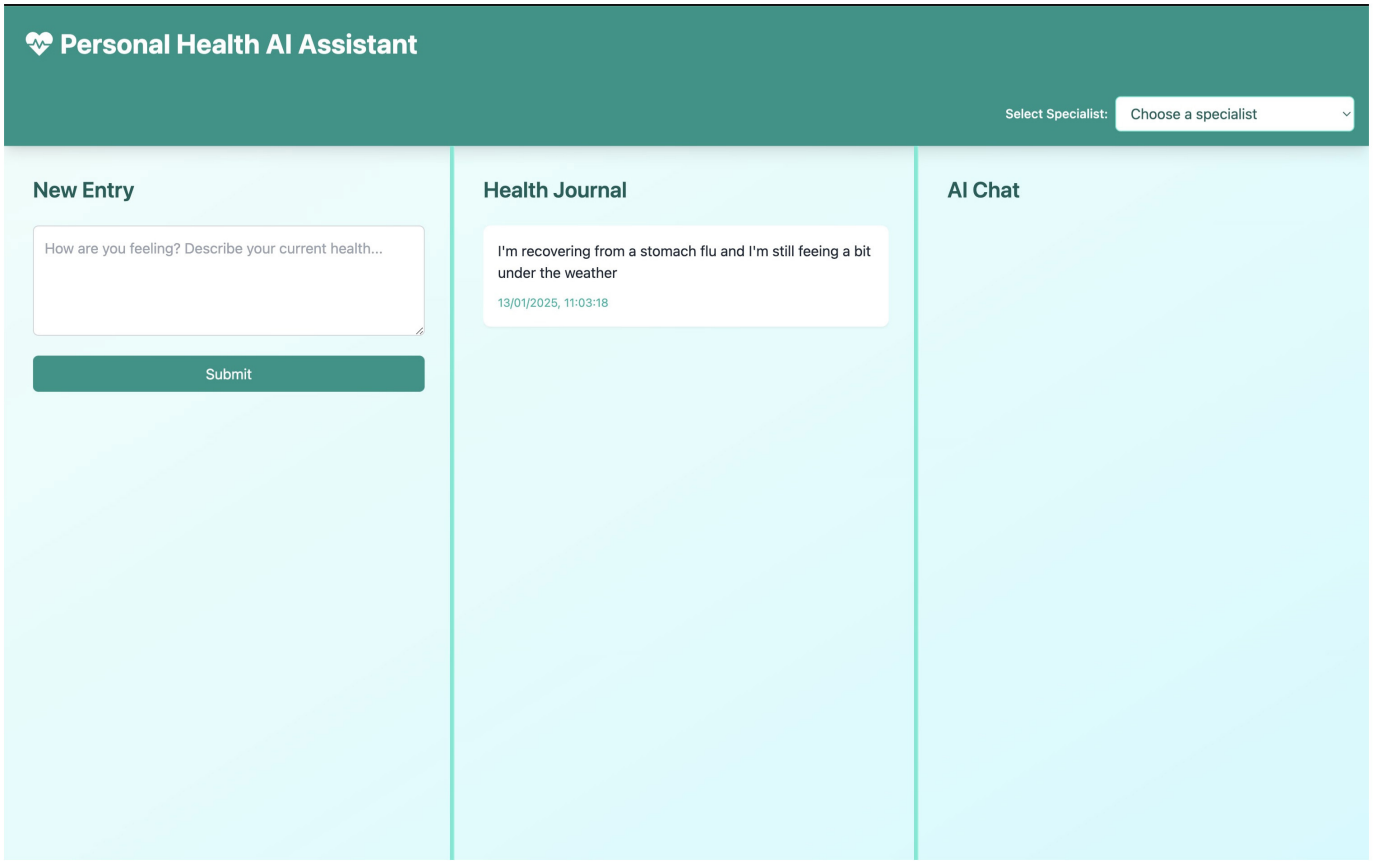
A health entry being recorded into the application's interface. **Left** shows the list of past entries, **middle** is for the new entry, and **right** is for AI interaction.

**Figure 3:**
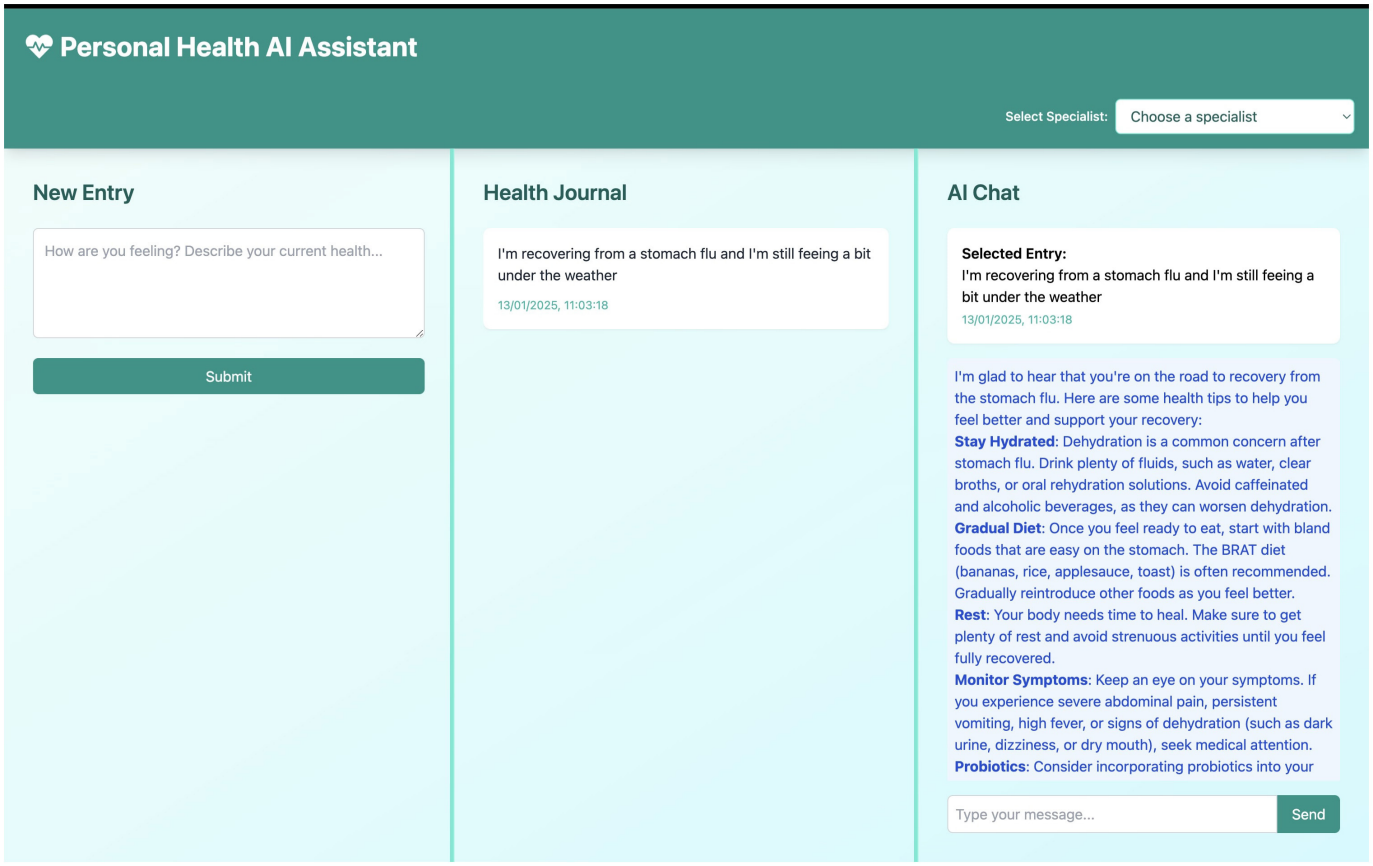
Response from an AI model (displayed in the **right**) based on the health entry made by the user in the **middle**.

**Figure 4:**
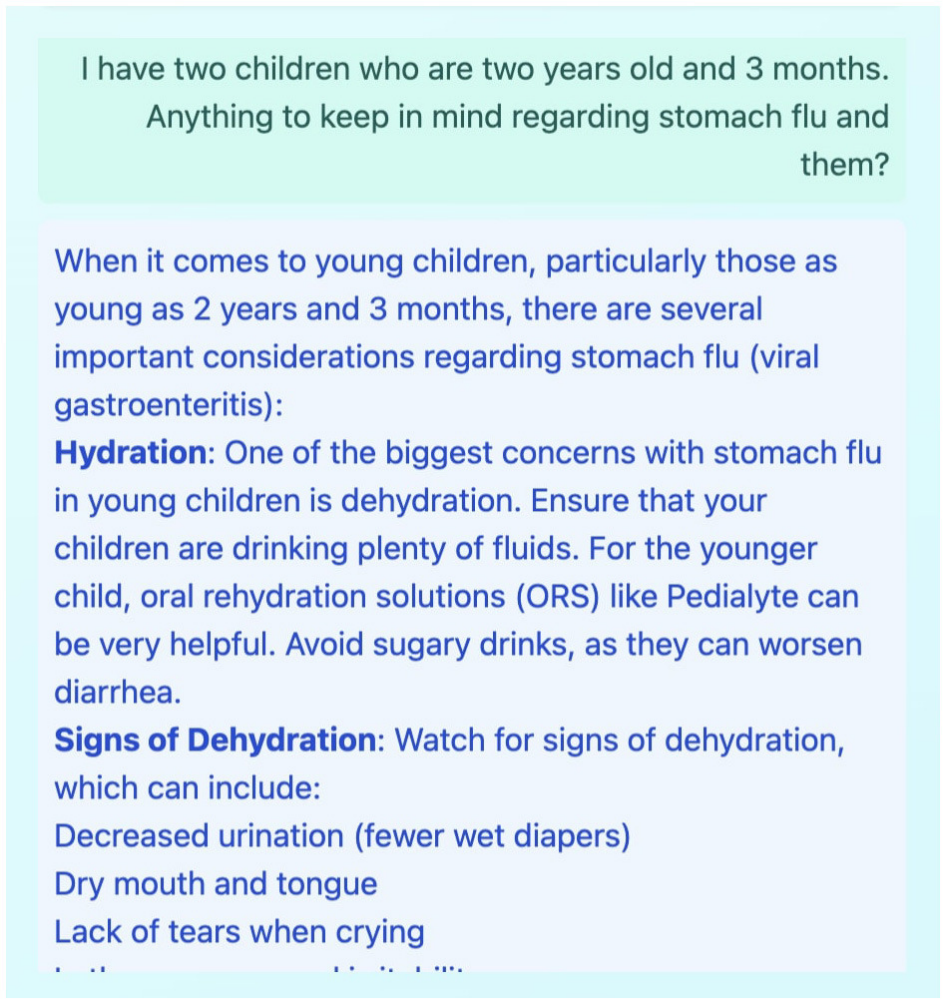
Example of a follow-up question posed by the user to the LLM in the AI Chat panel, based on the AI's initial response to a journal entry.

The interface of our functional prototype (illustrated in Figure 2) comprises three primary components designed to facilitate seamless interactions with the AI and capture user insights effectively:

- **New entry**: A freeform text field allows users to log their health status, symptoms, questions, or experiences. This flexible approach encourages natural language journaling, capturing the user's authentic voice and health journey in real time.
- **Health journal**: A chronological record of all entries enables longitudinal tracking of self-reported health data. This feature supports retrospective analysis by the user and can be compiled for sharing with healthcare providers as needed.
- **AI chat**: A contextual conversational interface leverages LLM capabilities to interpret user entries, offer personalized feedback, and reference relevant general health resources. An example of an AI response is shown in Figure 3, and a follow-up interaction is depicted in Figure 4.

The goal of the interface is to provide an intuitive way to record personal health information in a user-friendly format and to gain supportive feedback on one's health from an LLM.

#### 2.2.1 User profile

**Figure 5:**
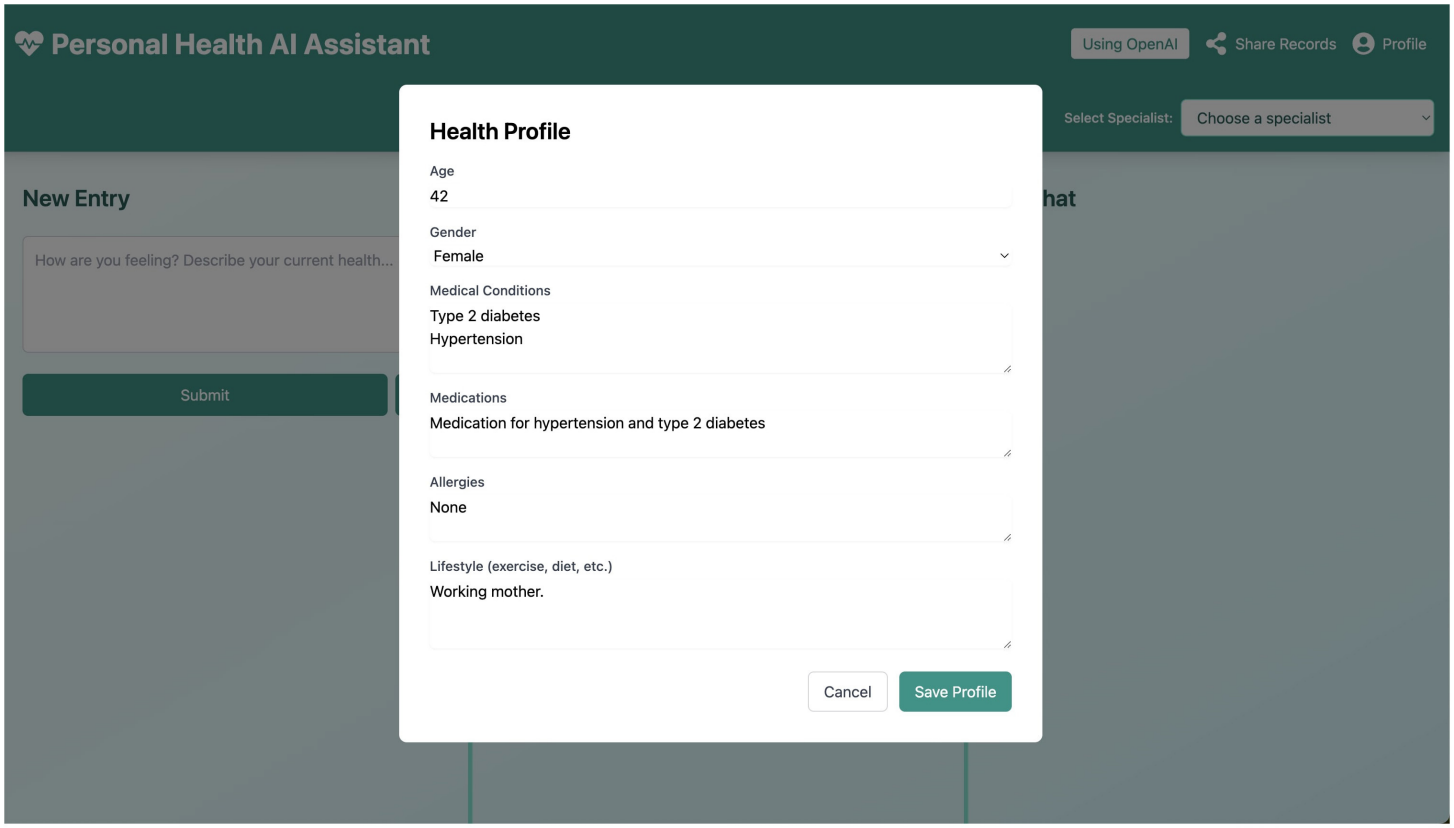
The User Profile interface, where users can input and store persistent health information to provide context for LLM interactions.

In the user profile section, shown in Figure 5, the user can add baseline information relevant to their medical history (e.g., chronic conditions, allergies, regular medications). This information is intended to be sent along with each journal entry to the LLM to provide consistent context. This is key for ensuring that specific, enduring health information is considered by the LLM when generating responses, beyond the content of individual entries.

#### 2.2.2 Send information to EHR

**Figure 6:**
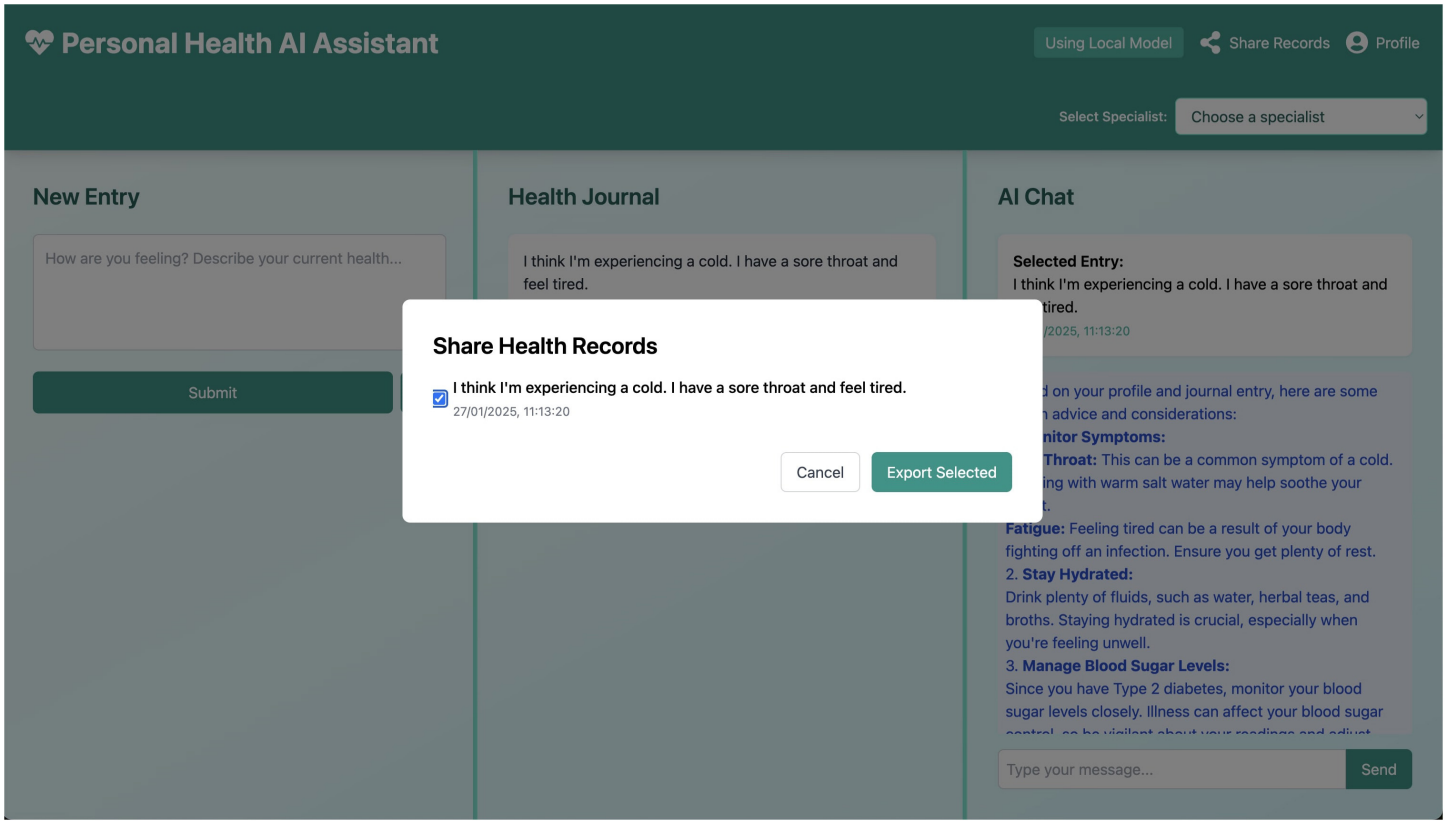
Conceptual interface for sharing or exporting health records, emphasizing user control over their data.

The user can, at any point, choose to download or compile information from their health journal, as illustrated conceptually in Figure 6. This data can then be shared with an EHR system or healthcare provider at the user's discretion. We prioritize an interface where the user has full control over their data. In the future, direct, secure integration with EHR systems could be explored, contingent on robust consent mechanisms and technical standards.

#### 2.2.3 Dictation

A dictation feature is included to improve ease of use, particularly for users who may find typing cumbersome or have mobility limitations. By pressing the dictation button, the user can verbally record a journal entry, which is then transcribed into text.

#### 2.2.4 Local models

A toggle is available in the interface, allowing the user to switch between cloud-based LLMs and (when available and configured) local LLMs running on the user's device. The availability of local LLMs is important for enhancing privacy and can aid in compliance with AI and healthcare regulations by keeping data processing on-device.

### 2.3 Strengths of the design

Several key strengths of the proposed journaling app underscore its potential for reshaping patient engagement:

- **Natural communication**: Daily journaling in natural language provides an intuitive pathway for patients to express their health concerns, promoting the capture of high-quality, nuanced data that might be missed in structured forms.
- **Integrated insights**: The AI leverages real-time context from the user's journal and profile, offering personalized suggestions and potentially highlighting trends or concerns for the user's reflection (not as a diagnostic tool).
- **Continuity of care support**: A comprehensive health journal, potentially summarized by AI, can facilitate more targeted and informed discussions with healthcare professionals, particularly for chronic conditions requiring ongoing monitoring.

In designing this prototype, we prioritized simplicity, transparency, and user empowerment. Although its clinical effectiveness requires future validation, these foundational elements lay the groundwork for exploring AI-driven journaling in broader healthcare contexts.

### 2.4 Technical overview of the functional prototype

The Personal Health AI assistant application is a desktop-based tool developed using Electron and React. It is a functional prototype designed as an AI-enhanced personal health record system, combining journal-keeping capabilities with AI-driven health insights and personalized informational responses. It is not intended for diagnostic or treatment purposes. All the code is available open source.

#### 2.4.1 Core technologies

The application's frontend is built with React 18, providing a modern, component-based structure for the user interface. Electron serves as the desktop framework, enabling cross-platform deployment (Windows, macOS, Linux) and access to native system functionalities. For AI integration, the prototype primarily utilizes the OpenAI API, with a design that accommodates potential future support for on-device models. UI/UX enhancements are implemented using Framer Motion for animations and React Icons for intuitive visual elements. Content rendering, particularly for AI-generated responses, uses React Markdown to display formatted text.

#### 2.4.2 Key features implementation

The **Journal Management** system allows users to create, store, and retrieve personal health journal entries. Entries are timestamped for chronological organization, support rich text for detailed record-keeping, and are automatically saved to ensure data integrity.

**AI Integration** is managed through API calls to an external LLM service (e.g., OpenAI). The application sends the current journal entry, along with relevant historical context from previous entries and the user profile, to the LLM. The system is designed for real-time analysis of these entries to generate context-aware informational insights and dynamic responses. Streaming responses are implemented to provide immediate feedback to the user. Prompt engineering techniques are employed to guide the LLM toward providing supportive and educational content, rather than medical advice, and to align responses with selected specialist modes.

The **Multi-Panel Interface**, as shown in Figures 2–4, is a core design element, featuring a responsive three-panel layout (journal list, current entry, AI chat) with resizable sections to suit user preferences and maintain a persistent layout.

**Healthcare Specialization** is implemented through pre-defined system prompts that tailor the LLM's responses. Users can select a mode (e.g., General Practitioner, Nutritionist, Mental Health support), which modifies the instructions sent to the LLM to frame its persona and the focus of its responses. This allows for a more structured organization of health data exploration and supports interaction using common medical terminology in a simplified manner.

The integrated **Chat System** provides a dedicated interface for AI interactions, with a persistent chat history linked to individual journal entries. It supports Markdown for formatted responses and real-time updates.

Native **Desktop Integration** via Electron allows for local data storage and backup, enhancing privacy. The user profile (Figure 5) allows users to input specific health information, which is stored locally and used as context for LLM interactions across sessions.

**Data Management** emphasizes local storage to ensure privacy, with data export capabilities (as conceptualized in Figure 6) allowing users to take their information. Security measures include prioritizing local-first architecture to minimize external data exposure. While full end-to-end encryption for local storage and secure API communication protocols are standard practices, the depth of their implementation in this early prototype is foundational, with recommendations for further hardening in any production-level system.

## 3 Results

### 3.1 Preliminary evaluation using user personas

To illustrate the versatility of the journaling application and to conduct an initial, qualitative assessment of its design and conceptual utility, we identified seven user personas. These personas represent diverse user needs and scenarios (Table 1). This method, a form of heuristic evaluation, helped us explore how different individuals might use the system to manage their unique health challenges and refine the prototype's features and interface based on anticipated user journeys. It is important to note that this persona-based evaluation is a preliminary step and not a substitute for real-world user testing or clinical validation.

**Table 1:** Personas and their anticipated usage of the healthcare journaling application.

**Persona**	**App usage scenario**	**Key app features supporting them**
**Anna, the Chronic Condition Manager (diabetes)**	Logs blood sugar levels, blood pressure, meals, and exercise. Asks the AI for general information on how diet affects her logged sugar levels. Considers sharing summarized logs with her GP.	• ✓ AI-driven contextual information based on entries (not medical advice) • ✓ Quick logging • ✓ Pattern highlighting (e.g., “You've logged high sugar after specific meals”) • ✓ Exportable summaries
**Johan, the Senior Tech Novice (arthritis)**	Uses voice-to-text to record daily pain levels and activities that exacerbate or alleviate symptoms. Asks AI for general tips on managing arthritis discomfort.	• ★ Simplified large-font interface option • ✓ Easy navigation • ✓ Voice-to-text input • ✓ AI providing general, evidence-based information on arthritis self-care • ✓ Tailored exercise guidance (general)
**Fatima, the expecting mother (pregnancy)**	Tracks pregnancy symptoms (e.g., nausea, fatigue), moods, and questions for her next prenatal appointment. Uses AI to get general information about common symptoms at her stage of pregnancy.	• ✓ Symptom tracking • ✓ AI providing educational resources on typical pregnancy progression • ✓ Suggestions for questions to ask her doctor.
**Lars, the fitness enthusiast (knee injury recovery)**	Monitors knee injury recovery by logging pain levels during specific exercises, mobility range, and rehabilitation milestones. Asks AI for general information on safe recovery practices.	• ✓ Logs pain levels during specific exercises, mobility range, and rehabilitation milestones for knee-injury recovery • ✓ AI provides general information on safe recovery practices • ✓ AI-driven analysis of recovery progress (e.g., “Pain levels during X exercise seem to be decreasing”) • ★ Mobility tracking • ★ Motivation through milestone logging
**Maja, the caregiver with chronic fatigue**	Logs energy levels, sleep quality, daily tasks, and stress triggers. Uses AI to reflect on patterns between activities and fatigue.	• ✓ Stress management techniques (general information) • ★ Personalized self-care reminders (user-set or AI-suggested based on patterns) • ✓ Insights for healthcare discussions (e.g., summarized fatigue patterns)
**Emma, the young adult with anxiety and depression**	Tracks mood, sleep patterns, medication adherence (if any), and reflections from therapy sessions. Uses AI for supportive dialogue and to reflect on gratitude or achievements.	• ✓ Secure journaling space • ✓ AI offering empathetic (but not therapeutic) responses • ✓ Coping strategy suggestions (general, evidence-based) • ✓ Trigger pattern identification • ★ Habit formation reminders (user-set)
**Peter, the middle-aged adult recovering from a heart attack**	Logs physical activity (e.g., daily steps), dietary changes, medication intake, and any recurring symptoms like chest discomfort or breathlessness. Tracks recovery milestones.	• ★ Medication reminders (user-set) • ✓ General lifestyle tips for cardiac health • ★ Recovery plan adherence tracking against user-set goals • ✓ Symptom logging for discussion with cardiologist

Each persona represents unique user scenarios, showcasing how the app's designed features aim to adapt to diverse health needs through AI-assisted self-monitoring and information provision. The “Key App Features” are functionalities of the prototype intended to support these uses. The Key App features includes both implemented features ✓ and suggested features ★. All features can be used without link to an EHR.

The personas typically represent individuals with moderate, often chronic, health concerns where increased insight and structured self-monitoring could be beneficial for patient well-being and self-management. For instance, Anna, managing diabetes, logs blood sugar levels and meals. The application, through its AI chat and journaling features, can support her by offering general information related to diabetes management based on her entries (e.g., a non-prescriptive discussion of how diet might impact blood sugar, based on established guidelines). Johan, a senior individual, might use voice-to-text to track arthritis pain and activities; the AI could then summarize these entries or provide general information about arthritis self-care strategies. Fatima can track pregnancy symptoms; the AI might offer general information about common pregnancy timelines or suggest when to consult a healthcare professional for specific (but non-emergency) concerns, based on her logged data. Emma, dealing with anxiety, can use the app to track mood and sleep, and the AI might provide supportive responses or suggest mindfulness exercises (without claiming to be therapy). These examples illustrate how the application is designed to support the psychological and informational aspects of managing health conditions, fostering patient autonomy.

The application serves a dual purpose: assisting the patient in managing their condition through informed self-reflection and data tracking, and potentially improving their understanding of their health. The journal entries, if shared by the patient, could also offer valuable longitudinal data for healthcare workers.

The “Key Features Supporting Them” column in Table 1 identifies specific, designed features of the application that are intended to address the needs of each persona. For example, “personalized health insights” refers to the AI's ability to comment on trends or patterns it might notice in the user's entries (e.g., “I see you've mentioned headaches more frequently this week”), while “actionable advice” would be limited to general wellness tips or suggestions to consult a professional, not specific medical directives. The application can produce the described functionalities (journaling, AI feedback based on entries and profile) independently of any EHR linkage. EHR integration is a potential future data-sharing feature, entirely at the user's discretion.

### 3.2 Use case insights from persona-based evaluation

Working through the persona-based scenarios provided valuable insights that informed the iterative design of the prototype. For example, the need for a **speech-to-text** dictation feature became evident when considering personas with limited mobility (like Johan) or those who might prefer verbal input for convenience. The **user profile** (Figure 5) was enhanced to allow for more structured input of chronic conditions or baseline health data, a need highlighted by personas like Anna (diabetes) or Peter (post-heart attack), ensuring the AI has relevant standing context beyond individual entries.

This persona-based approach served as a useful first step in UX testing for a potential medical application in a safe yet relatively realistic manner. By adopting the needs of these personas and testing the application's design against their anticipated interactions, we could simulate how real users might engage with the system without involving actual patients at this early developmental stage. In the fast-moving field of AI, such qualitative, simulation-based evaluations can be a helpful method to rapidly assess new AI technology concepts and refine designs before undertaking more resource-intensive user studies, while minimizing risks.

### 3.3 Risks of health journaling with AI and mitigation strategies

While AI-assisted health journaling offers potential benefits, it is crucial to acknowledge and address associated risks.

#### 3.3.1 Risks related to AI tools

A primary risk associated with LLMs is their propensity for “hallucination”–generating plausible but incorrect or nonsensical information (Lewis et al., 2021). In healthcare, this could lead to users receiving inaccurate health information. Mitigation strategies include:

- **Retrieval augmented generation (RAG):** while not fully implemented in this prototype, future iterations could use RAG to ground LLM responses in curated, reliable medical knowledge bases, thereby reducing hallucinations and improving factual accuracy.
- **Clear disclaimers and user education:** users must be explicitly and repeatedly informed that the AI is not a medical professional, does not provide medical advice, diagnosis, or treatment, and that its information may not always be accurate or complete. They should always consult qualified healthcare providers for medical concerns.
- **Prompt engineering:** carefully designed prompts instruct the LLM to avoid making definitive medical statements, to encourage users to see healthcare professionals, and to cite sources if RAG is implemented.
- **Structured Evaluation:** Ongoing evaluation of the LLM's output quality, accuracy, and safety is essential.

Another risk is **LLM bias**, stemming from biases present in their training data. This could lead to skewed information or advice, particularly for underrepresented demographic groups (Nassiri and Akhloufi, 2024). Mitigation involves striving for diverse datasets in model training (though often outside the app developer's control for foundational models) and being transparent about the potential for bias.

**Anthropomorphizing AI tools** (Deshpande et al., 2023) can lead to over-reliance, where users might substitute AI interaction for consultation with human healthcare providers. This could delay necessary medical attention and potentially worsen health outcomes. Clear communication about the AI's role as a supportive tool, not a replacement for professional care, is critical.

To implement such systems safely in clinical practice, a **human-in-the-loop** approach is often recommended (Jackson and Pinto, 2024). While our application is patient-facing, if data is shared with clinicians, it should be presented as supplementary information, with clinicians maintaining ultimate diagnostic and treatment responsibility. A clinician interface that summarizes journal entries could facilitate this, but the raw, subjective nature of journaling must be understood by the clinician.

#### 3.3.2 Other risks inherent to health journaling

Health journaling focuses on the individual's subjective experience. While valuable, the quality and completeness of health data can be limited by this open-ended format. Important structured data might be omitted, or symptoms misinterpreted by the user. Furthermore, journaling, even with AI feedback, might provide a **false sense of security** or, conversely, induce **health anxiety** if users misinterpret AI responses or focus excessively on minor symptoms. These are limitations of an open journaling format compared to structured questionnaires for specific disorders. The AI should be designed to be reassuring yet cautious, consistently guiding users toward professional consultation when appropriate.

#### 3.3.3 Regulatory and data privacy risks

Handling sensitive personal health information (PHI) necessitates strict adherence to data privacy regulations like GDPR in Europe or HIPAA in the US. Our local-first architecture is a step toward compliance, but secure data handling, robust encryption (at rest and in transit for API calls), clear consent mechanisms for data use (especially if sharing with third-party LLMs), and data breach protocols are paramount. The evolving nature of AI regulation (e.g., the EU AI Act) also requires ongoing attention to ensure compliance, especially concerning transparency, risk management, and data governance for AI systems.

### 3.4 Summary of findings from prototype development and persona evaluation

This paper introduces a functional prototype of a novel AI-driven journaling application designed to enhance patient engagement via a natural language interface. The system enables patients to document their health experiences and receive real-time, context-aware informational feedback from an LLM. The core aim is to bridge the gap between the qualitative richness of patient narratives and the need for structured self-monitoring, focusing on user experience, ethical considerations, and potential clinical integration.

Key aspects of the prototype include a three-panel interface for journaling, AI dialogue, and longitudinal health tracking, with specialized modes to simulate interaction with different healthcare expert perspectives. Persona-based qualitative evaluations suggest the system has the potential to support health literacy by providing explainable AI responses (within the bounds of non-medical advice) while being architected for data localization and privacy (e.g., local storage, option for local LLMs).

Based on this development work, we propose five design principles for patient-centric AI health tools: (1) decoupling core functionality from LLM dependencies to ensure basic utility and facilitate regulatory adaptation; (2) layered transparency in AI outputs, clearly stating limitations and sources; (3) adaptive and granular consent for data sharing, empowering users with control; (4) clinician-facing summarization tools if data is to be shared with healthcare providers, translating narrative data into concise insights; and (5) a compliance-first architecture, prioritizing data security and regulatory adherence from the outset.

By aiming to transform unstructured patient narratives into more understandable patterns through natural language processing, this AI-assisted journaling approach shows potential as a middleware layer in healthcare ecosystems. It seeks to empower patients as active partners in their care while preserving the necessity of clinical oversight. Future research must involve rigorous user trials to evaluate impacts on care continuity, patient-provider communication, and long-term health outcomes.

## 4 Discussion

The prototype journaling application presented in this study establishes a dynamic interface between patient-reported health observations and AI-driven informational feedback. By leveraging the journaling paradigm, the system encourages patients to record their everyday health experiences–symptoms, lifestyle choices, psychosocial factors–in real-time and in their own words. Unlike the often static and structured data in electronic health records (EHRs), this approach aims to capture a richer, more nuanced view of patients' lived experiences. Existing literature highlights the clinical value of such patient-centered narratives in uncovering insights often missed by conventional EHR systems (Malgaroli et al., 2025; Azimi et al., 2025). The proposed system thus offers a promising avenue for potentially bridging some communication gaps between patients and providers, and fostering patient self-understanding.

### 4.1 Enhanced patient engagement and data quality

A distinguishing feature of this prototype is its integration of LLMs to provide immediate, context-aware feedback on journal entries. This capability is intended not only to enhance user engagement but also to encourage patients to reflect on and provide richer, more detailed health data. By offering real-time, tailored informational suggestions or insights (e.g., noting patterns, providing general health education related to logged topics), the AI may help users identify early warning signs or access relevant educational content, thereby fostering proactive health management. However, the success of such a system depends heavily on the perceived reliability, trustworthiness, and utility of the AI's responses. Transparency mechanisms, such as clearly displaying the AI's confidence levels (if available), referencing credible health guidelines (if RAG is implemented), and explicitly clarifying the non-diagnostic, non-advisory nature of AI outputs, are essential to build and maintain user trust and manage expectations.

The journaling approach also aligns with principles of reflective practice, where patients are encouraged to contemplate their health status over time. This process can deepen user engagement, promote self-awareness, and indirectly enhance clinical encounters by enabling patients to articulate their health concerns more effectively during consultations. Nevertheless, careful attention must be paid to balancing engagement with user effort; overly complex interfaces, unhelpful, or unclear AI responses risk diminishing sustained use.

### 4.2 Comparison with existing solutions

While various health and wellness apps exist, many focus on specific niches like fitness tracking (e.g., Fitbit, Strava), diet logging (e.g., MyFitnessPal), or mental wellness journaling (e.g., Daylio, Woebot). Some mental health apps incorporate rule-based or basic AI chatbots for support (Abd-alrazaq et al., 2023). The novelty of our proposed system lies in its attempt to create a comprehensive, multi-purpose health journal powered by advanced LLMs, capable of understanding and responding to a wide range of unstructured natural language inputs about diverse health topics. Key differentiators include:

- **Broad-spectrum input:** designed to handle diverse health narratives beyond single-issue tracking.
- **Deep LLM integration:** aims for more nuanced, context-aware feedback than simpler chatbots.
- **Three-panel UX:** a specific interface designed for concurrent journaling, AI interaction, and historical review.
- **Emphasis on local processing potential:** forward-looking design for on-device LLMs to maximize privacy.
- **Reflective self-monitoring focus:** encourages active interpretation of health data through AI dialogue.

Compared to clinical EHR patient portals, which are primarily for accessing official records or secure messaging, our tool is designed for continuous, patient-driven narrative input and AI-facilitated self-reflection. It is a personal tool first, with optional sharing capabilities.

### 4.3 Ethical considerations, data security, and LLM limitations

The local-first architecture of the prototype is a foundational step in minimizing data exposure by storing journal entries on users' devices. However, the implementation of robust end-to-end encryption for any data in transit (e.g., to cloud LLMs if local models are not used) and at rest, along with strong privacy-preserving measures, is critical. Mechanisms for dynamic user consent and granular control over data sharing will be pivotal. The system's architecture supports these principles by decoupling journaling functionality from AI components, allowing users to retain autonomy.

The limitations and risks of LLMs, including hallucination, bias, and the potential for misuse, must be prominently addressed as detailed in the “Risks” section. Transparency about these limitations is key. For instance, the AI should not be presented as an omniscient medical expert but as an informational assistant. The risk of users developing an over-reliance on the AI or misinterpreting its outputs as definitive medical advice is significant and requires ongoing mitigation through UI design, disclaimers, and user education.

From a regulatory perspective, developers must navigate evolving healthcare compliance requirements (e.g., GDPR, HIPAA, EU AI Act). By designing the application to operate effectively as a standalone journaling tool, with AI functionality as an optional enhancement, the system provides flexibility. Clear disclaimers about the advisory (or rather, informational and non-advisory) nature of AI-generated responses are necessary to manage liability.

### 4.4 Comparison to available solutions

There are several companies working on health journaling both for mental health (Mindsera, 2025) and in the medical domain (Tambool LLC, 2024, 2025). These applications work as standalone applications where users can journal about health with an AI system, that is not fully specified but is likely an LLM. We found no open source system similar to ours and no system that combined web, native and standalone applications for AI health journaling. In comparison to these systems our system is more transparent, providing open source code and description of the systems functioning.

### 4.5 Future research directions

While the initial prototype development and persona-based evaluation offer conceptual validation, this work is at an early stage. Rigorous future studies are essential. Specifically, clinical trials and comprehensive user experience research should:

- Evaluate the system's efficacy in improving specific health outcomes, such as adherence to care plans, accuracy in symptom tracking for certain conditions, or impact on health literacy.
- Assess usability, engagement, and perceived utility across diverse patient populations, accounting for varying digital literacy levels, cultural contexts, and access to technology.
- Explore the system's capacity for long-term engagement and its impact on user behavior and patient-provider communication over extended periods.
- Investigate the psychological impact of long-term AI interaction for health journaling, including potential for increased anxiety or false reassurance.
- Technically, further research into robust RAG systems with curated medical knowledge is needed to improve AI reliability.

Additionally, future work should explore best practices for integrating user-generated journaling data with EHR systems while preserving privacy and ensuring data integrity. Approaches such as federated learning or secure multi-party computation could be considered for collaborative data analysis without direct sharing of raw patient data. By potentially embedding summarized, patient-consented journaling insights into clinical workflows, the system could enhance care coordination, support shared decision-making, and streamline patient history intake.

### 4.6 Open source and collaborative approach

The Health Journaling application is an open-source project. We actively encourage participants to contribute to the development of the tool.

### 4.7 Implications for healthcare systems

The findings from this prototype development highlight the potential of journaling-based AI interfaces to address certain gaps in healthcare communication and patient self-management. By facilitating continuous, context-aware patient engagement, such tools can support self-management for chronic conditions and complement traditional care models. The ability to integrate personalized AI feedback into patient-driven narratives positions these systems as potentially transformative instruments for preventive care and chronic disease management, provided they are developed and deployed responsibly.

However, scalability and sustainability will depend on a robust infrastructure that integrates ethical AI design with adaptable user interfaces and clear pathways for clinical integration where appropriate. As LLM technology evolves, periodic re-evaluation of features, functionalities, and the underlying AI models will be necessary to ensure their ongoing safety, relevance, and alignment with user needs and ethical standards.

In summary, this study provides a foundational step toward developing AI-powered journaling systems that prioritize user empowerment, data privacy, and potential healthcare integration. While significant challenges in terms of AI reliability, ethics, regulation, and practical implementation remain, these tools hold the potential to reshape patient-provider interactions and foster a more holistic, data-driven, and patient-centered approach to healthcare.

### 4.8 Limitations

A key limitation in this work is a lack of human evaluation. Relying on personas to guide user centric design is important but should be seen as a first step where necessary changes in design needs to be made when the system in later stages are evaluated by humans. Still we believe that work like this is important, since the fast pace of progress in AI and the substantial regulatory burdens for clinical evaluations requires creative solutions for keeping track with technology while ensuring regulatory compliance and ensuring no harm to patients. By focusing on design and user centric needs and building open source software, we can keep track of AI progress while maintaining safety.

## 5 Conclusion

The emergence of LLM-driven journaling applications underscores a pivotal evolution in patient engagement, where AI can become an active partner in an individual's ongoing health management journey. By merging thoughtful design that emphasizes transparency, patient-centered interfaces, and ethical data handling, these tools have the potential to serve as a new layer of healthcare communication and self-understanding that transcends traditional electronic records. Our functional prototype and conceptual framework exemplify how a journaling platform—augmented with real-time AI responses—can empower patients to understand their health more holistically and potentially share meaningful, contextualized insights with care providers.

While this work represents an early-stage proof of concept and has not yet undergone clinical validation or large-scale user testing, it highlights the considerable potential of LLM-powered journaling to fill critical gaps in patient-AI interactions and patient-driven health data generation. Future research must prioritize rigorous user testing across diverse populations, cross-cultural evaluations, and clinical trials to establish efficacy, safety, and the overall impact on health outcomes and patient experience. Looking ahead, we envision patient journaling applications, if developed and implemented responsibly, as catalysts for more equitable, user-friendly, and personalized healthcare systems, ultimately enhancing self-management capabilities and augmenting clinical decision-making worldwide.

## Data Availability

The datasets presented in this study can be found in online repositories. The names of the repository/repositories and accession number(s) can be found below: https://github.com/BirgerMoell/health-journal/.
